# The impact of histopathology on prognosis of squamous cell carcinoma of the larynx: can we do better?

**DOI:** 10.1007/s00428-025-04082-w

**Published:** 2025-03-27

**Authors:** Nina Zidar, Lester D. R. Thompson, Abbas Agaimy, Göran Stenman, Henrik Hellquist, Alfons Nadal, Antti A. Mäkitie, Fernando López, Primož Strojan, Alfio Ferlito

**Affiliations:** 1 Faculty of Medicine, Institute of Pathology, University of Ljubljana, Korytkova 2, 1000 Ljubljana, Slovenia; 2 Head and Neck Pathology Consultations, Woodlands Hills, CA USA; 3 Comprehensive Cancer Center (CCC) Erlangen-EMN, Institute of Pathology, University Hospital, Friedrich-Alexander University Erlangen-Nürnberg (FAU), Erlangen, Germany; 4 Department of Pathology, Sahlgrenska Center for Cancer Research, Sahlgrenska University Hospital, University of Gothenburg, Gothenburg, Sweden; 5 Department of Biomedical Sciences and Medicine, ABC-RI, University of Algarve, Faro, Portugal; 6 Department of Cellular Pathology, Northern Lincolnshire and Goole NHS Foundation Trust, Lincoln, UK; 7 Department of Pathology, Hospital Clinic, Barcelona, Spain; 8 Department of Basic Clinical Practice, School of Medicine, Universitat de Barcelona, Barcelona, Spain; 9 Department of Otorhinolaryngology-Head and Neck Surgery, Research Program in Systems Oncology, Helsinki University Hospital, University of Helsinki, Helsinki, Finland; 10 Department of Otolaryngology, ISPA, IUOPA, CIBERONC, Hospital Universitario Central de Asturias, University of Oviedo, Oviedo, Spain; 11 Department of Radiation Oncology, Institute of Oncology, Ljubljana, Slovenia; 12 University of Udine School of Medicine, Udine, Italy

**Keywords:** Larynx, Squamous cell carcinoma, Prognosis, Human papillomavirus, Biomarkers, Disease progression, Precision medicine, Lymphocytes, Tumour-infiltrating, Morbidity

## Abstract

Despite decades of progress, laryngeal squamous cell carcinoma (SCC) is still associated with significant morbidity and mortality worldwide. Additional biomarkers are needed to apply precision medicine and predict the clinical course. We reviewed and summarised routinely reported histopathologic features (e.g. subtypes of laryngeal SCC) along with promising potential biomarkers not yet routinely assessed using international guidelines. These include extra- vs intratumoural vascular and perineural invasion, tumour budding, depth of invasion, and tumour-infiltrating lymphocytes. We also address the problem of specimen quality and type (open approach vs endoscopic surgery) and the related limitations. High-risk human papillomavirus infection is another controversial issue to be discussed, being rare in laryngeal SCC, with an indeterminate prognostic significance and less reliable p16 overexpression as a surrogate marker of HPV infection. Further studies are warranted to address the applicability and to see which of the described parameters may help to better stratify patients with laryngeal SCC and should therefore be included in the pathology report.

## Introduction

Squamous cell carcinoma (SCC) of the head and neck is the sixth most prevalent cancer worldwide, accounting for 5% of all new cancers, and more than 200,000 new cases annually [1, 2]. It is a heterogeneous group of tumours that vary according to aetiology, anatomic subsite, and histologic subtype. The larynx is a commonly affected subsite, along with the oral cavity, lip, and pharynx, accounting for 25–30% of all head and neck cancers [1–3]. The incidence and mortality rates of laryngeal SCC (LSCC) have significantly decreased over the past three decades in most countries, while in some countries in East Asia and North Africa, increasing incidence and mortality rates have been observed [3, 4]. LSCC is strongly associated with cigarette smoking and alcohol intake, with synergistic effects [5, 6]. Gastroesophageal/laryngo-pharyngeal reflux and human papillomavirus (HPV) are minor aetiologic factors in LSCC [7, 8].

Significant progress has been made in head and neck pathology diagnostic refinement during the last decades, mostly based on new discoveries of the genetic background of tumours, resulting in new entities and more precisely defined diagnostic, prognostic, and predictive factors. This progress, together with new therapeutic approaches, has resulted in more personalised precision treatment, although less so in LSCC than in several other tumour types. For this reason, new prognostic and predictive biomarkers are needed in LSCC. In this review, we summarise the current knowledge of the pathology of LSCC, how it impacts the prognosis, and discuss possible new directions for improvement in prognostication.

## Squamous cell carcinoma of the larynx and its subtypes

The vast majority of LSCC is conventional type SCC, accounting for more than 90% of cases. According to the latest WHO Classification of Head and Neck Tumours [9], the remaining subtypes include verrucous carcinoma, basaloid SCC, papillary SCC, spindle cell SCC, adenosquamous carcinoma, and lymphoepithelial carcinoma, recognising that other very uncommon subtypes also develop, such as NUT carcinoma [10–12], adenoid SCC [13, 14], and carcinoma cuniculatum [15, 16]. These subtypes are true clinicopathologic entities, with prognostic significance and known differential diagnoses [17]. Several tumours considered in the differential diagnoses of LSCC subtypes show a canonical genetic background (e.g. salivary gland tumours, sarcomas, NUT carcinoma), with detection helpful in ambiguous cases.

*Verrucous carcinoma* (VC) is a subtype of well-differentiated SCC that lacks both the cytologic features of malignancy and infiltrative invasive borders. It is characterised by lateral spread and broad, pushing invasion below the level of the adjacent epithelium. It can cause extensive local destruction if left untreated. Pure VC does not metastasize [18]. The larynx is the second most common location of VC in the head and neck after the oral cavity. Aetiologically, it is associated with smoking. There is no evidence of transcriptionally active high-risk HPV in VC [19–21].

**Fig. 1:**
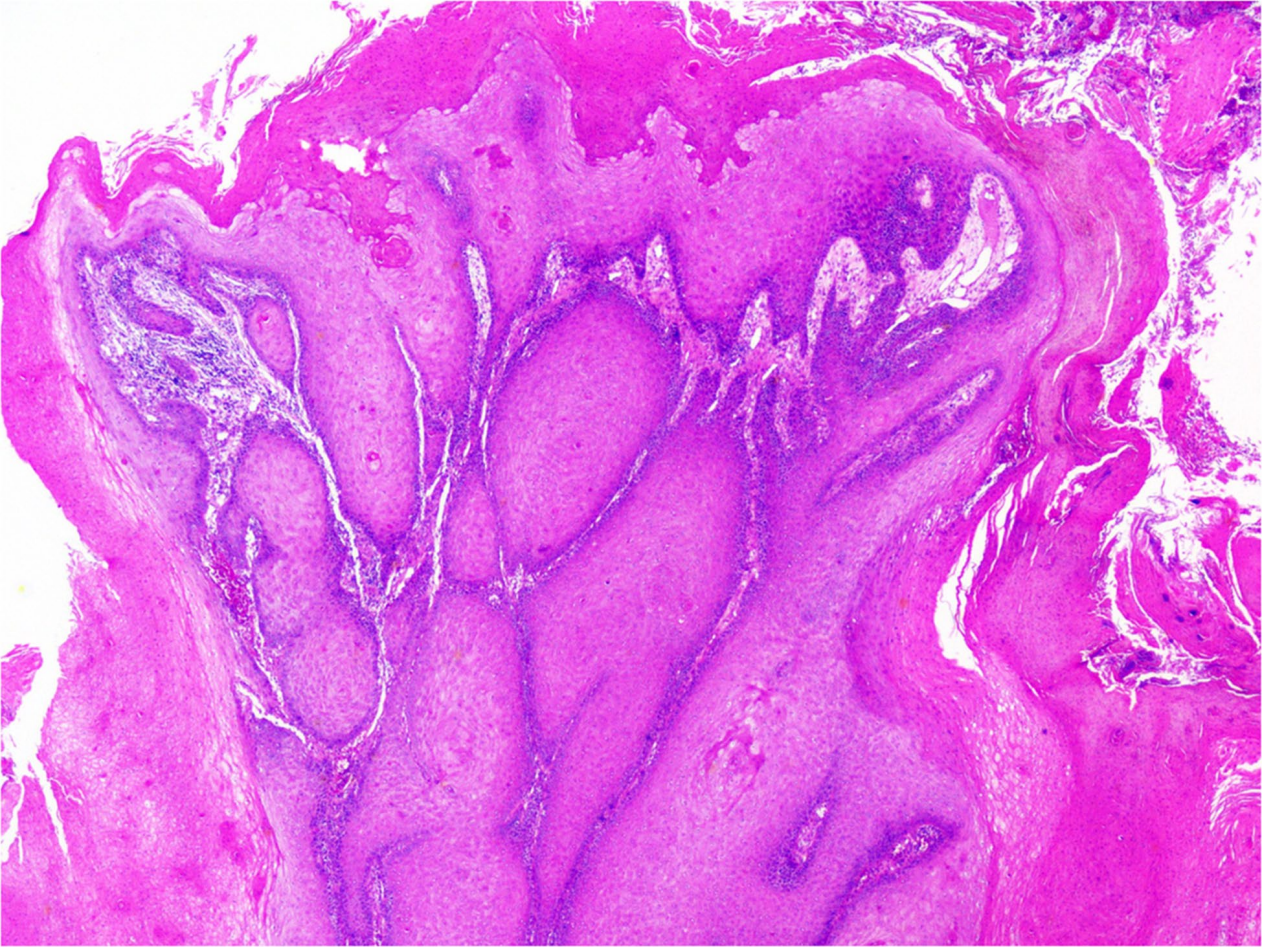
Verrucous carcinoma: projections and invaginations of well-differentiated squamous epithelium with marked surface keratinisation, invading the subjacent stroma with well-defined pushing border

Macroscopically, VC usually presents as a large, broad-based exophytic tumour with a whitish granular shaggy surface. On the cut surface, it is firm or hard, tan to white, and may show keratin-filled surface clefts [22]. Microscopically, VC consists of thickened undulating projections and invaginations of well-differentiated squamous epithelium with marked surface keratinisation (“church-spire” keratosis) (Fig. 1). The squamous epithelial cells in VC lack the usual cytologic criteria of malignancy. They are significantly larger than those seen in the benign mimics. Image analysis can be helpful in the discrimination, since laryngeal VC cells have a mean size larger than 300 µm^2^ and laryngeal papilloma cells less than 250 µm^2^ [23]. Mitoses are rare and confined to the basal and suprabasal layers. VC invades the subjacent stroma with well-defined pushing rather than infiltrative borders, below the level of the surrounding epithelium. The pushing borders are often surrounded by a dense inflammatory infiltrate. If VC harbours areas of conventional SCC, it is referred to as a hybrid (mixed) tumour and should be treated as conventional SCC [22]. However, Patel et al. [24] reported that VC with dysplasia or minimal invasion, defined as VC with SCC less than or equal to 2 mm in depth, does not affect the prognosis and behaves as VC.

The histologic confirmation of VC can be extremely difficult, particularly in small biopsy specimens and can significantly delay diagnosis and treatment. VC is characterised by a high frequency of initial misdiagnosis; Orvidas et al. [22] reported that 16 of 31 patients (51.6%) had received an incorrect initial diagnosis of a benign lesion. This emphasises the need for close cooperation between the pathologists and clinicians in order to establish a correct diagnosis. An adequate, full-thickness biopsy specimen must be taken, when a clinician suspects VC. Moreover, multiple biopsies or even an open biopsy at the time of surgery may be needed to confirm the diagnosis of VC or rule out a conventional SCC component in VC.

The differential diagnoses include verrucous hyperplasia which lacks evidence of invasion and conventional SCC which shows clear pleomorphism and an irregular infiltrative growth with a desmoplastic stromal reaction [18].

VC has a significantly better prognosis than conventional SCC, with reported overall survival rates of 80–95% [25, 26]. To date, no specific molecular findings have been reported.

*Basaloid SCC* (BSCC) is a subtype of SCC with prominent basaloid morphology, squamous differentiation, and aggressive biologic behaviour [27]. In the larynx, it is not associated with HPV infection [28, 29].

**Fig. 2:**
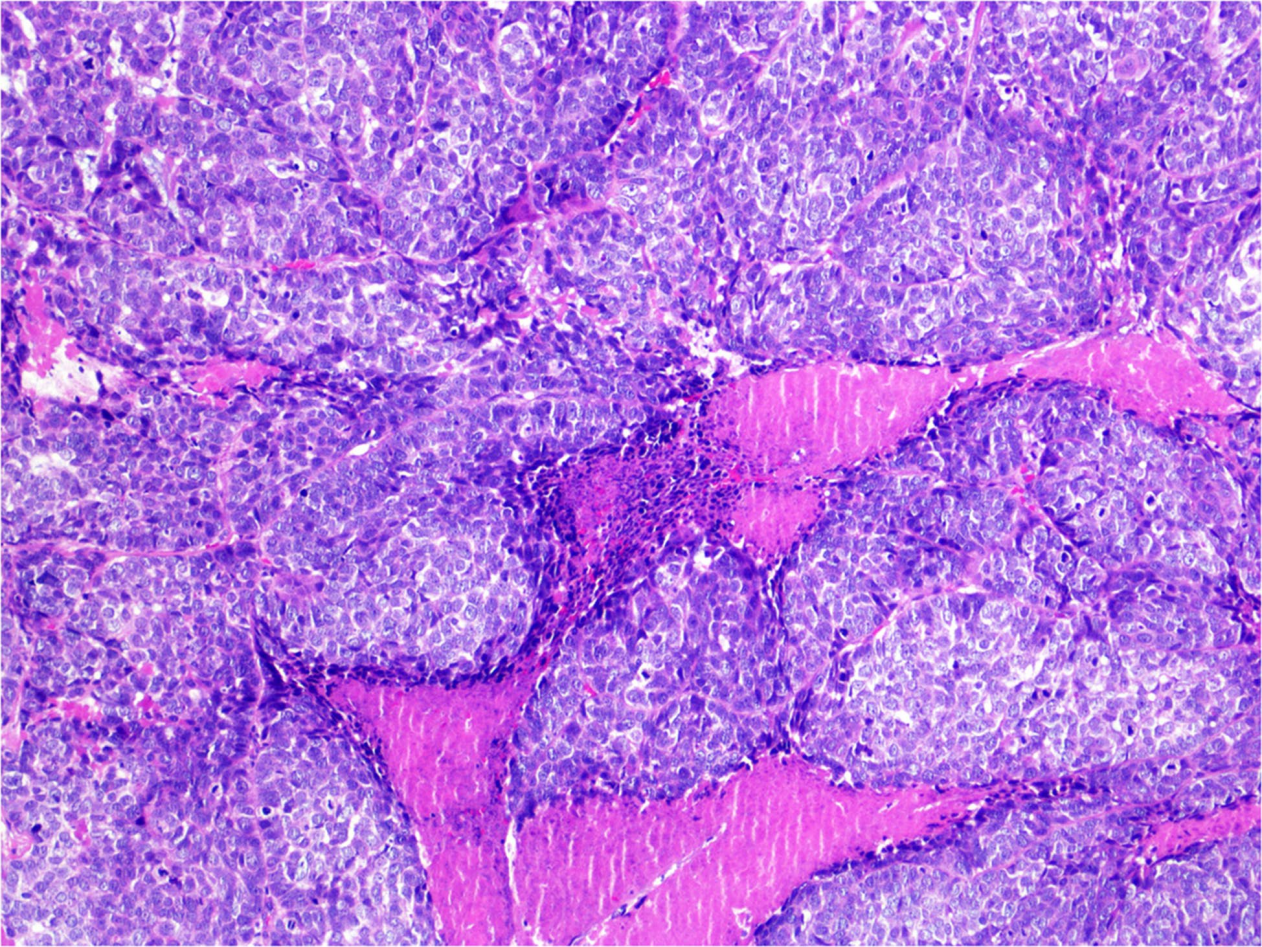
Basaloid squamous cell carcinoma: closely packed basaloid cells, with hyperchromatic nuclei and scant cytoplasm, with a lobular configuration

Microscopically, BSCC is composed of closely packed basaloid cells, which are small, with hyperchromatic nuclei with or without nucleoli, and scant cytoplasm (Fig. 2). It is almost always associated with a SCC component, which can occur either as an in situ or invasive SCC. The tumour grows in a solid pattern with lobular configuration and frequent peripheral palisading of nuclei. Large central comedo necrosis is frequent. The stroma is often myxoid, with focal hyalinisation or basal lamina formation [27, 30, 31]. BSCC expresses squamous markers (e.g. p40, p63, CK5/6) and lacks neuroendocrine markers (synaptophysin, chromogranin, INSM1) as well as S100 and TTF1 but may express SOX10, CD117 and MYB [28].

The differential diagnoses include neuroendocrine carcinoma, NUT carcinoma, and HPV-associated SCC extending from the oropharynx, which has a better prognosis than basaloid SCC. BSCC is distinguished from neuroendocrine carcinoma by squamous markers and lack of neuroendocrine markers. NUT carcinoma may look similar but is additionally positive for NUT immunohistochemistry. Some HPV-associated oropharyngeal SCCs demonstrate prominent basaloid features but behave much more indolently and should be distinguished from BSCC. Accordingly, direct detection assays for high-risk HPV must be performed on laryngeal BSCC that also involves the oropharynx [27, 28].

The prognostic significance of laryngeal BSCC is controversial. Some studies suggest comparable outcomes to conventional SCC [32, 33], whereas others indicate that in the larynx, BSCC is more aggressive than conventional SCC [34, 35].

**Fig. 3:**
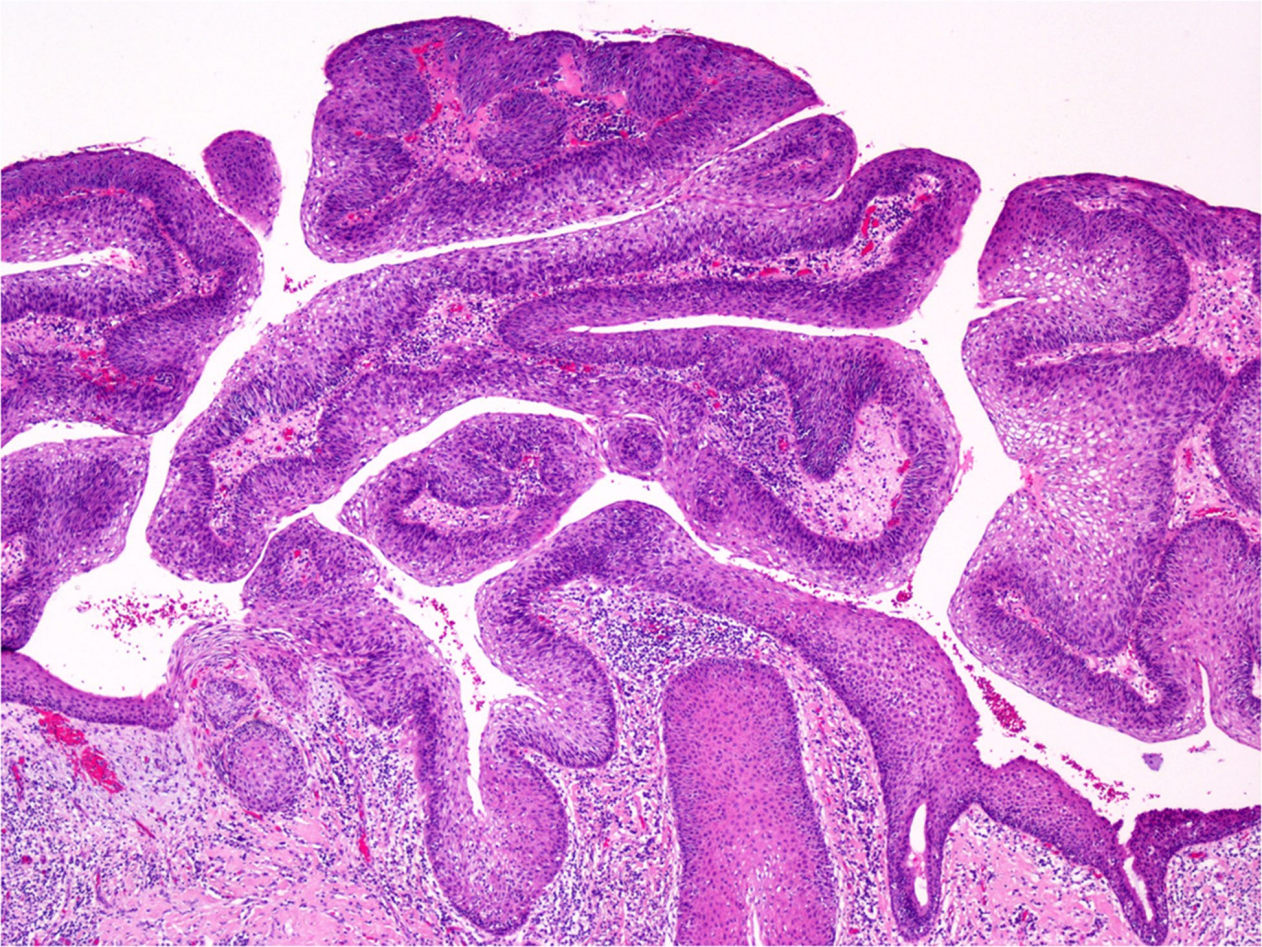
Papillary squamous cell carcinoma: exophytic growth, composed of papillae covered by atypical squamous epithelium, without invasion

*Papillary SCC* is characterised by exophytic growth and is composed of papillae covered by atypical stratified squamous or immature basaloid epithelium (Fig. 3) [36, 37].

It can be associated with high-risk HPV infection, but it is not clear whether or not the presence of HPV affects the prognosis [38–40]. Invasion may be difficult to prove, particularly in small biopsy specimens, and usually consists of irregular nests of non-keratinising SCC. Papillary SCC must be distinguished from VC, which is usually keratinising and lacks atypia, and from papilloma, which typically does not show atypia, while atypia is always present in papillary SCC [36, 37, 41]. Because of the exophytic growth, papillary SCC is usually diagnosed at an early stage and has a better prognosis than conventional SCC [36, 38–40].

*Spindle cell carcinoma* (SpCC) is composed of spindle and/or epitheloid pleomorphic cells, usually with a conventional SCC component [42]. There is evidence that epithelial-mesenchymal transition is the most likely pathogenetic mechanism underlying the transition of neoplastic epitheloid cells to neoplastic spindle cells [43, 44]. The larynx and oral cavity are the most frequent locations of SpCC in the head and neck. Aetiologically, SpCC is associated with cigarette smoking, alcohol abuse, and previous irradiation, developing 1.2 to 16 years after exposure [45, 46].

**Fig. 4:**
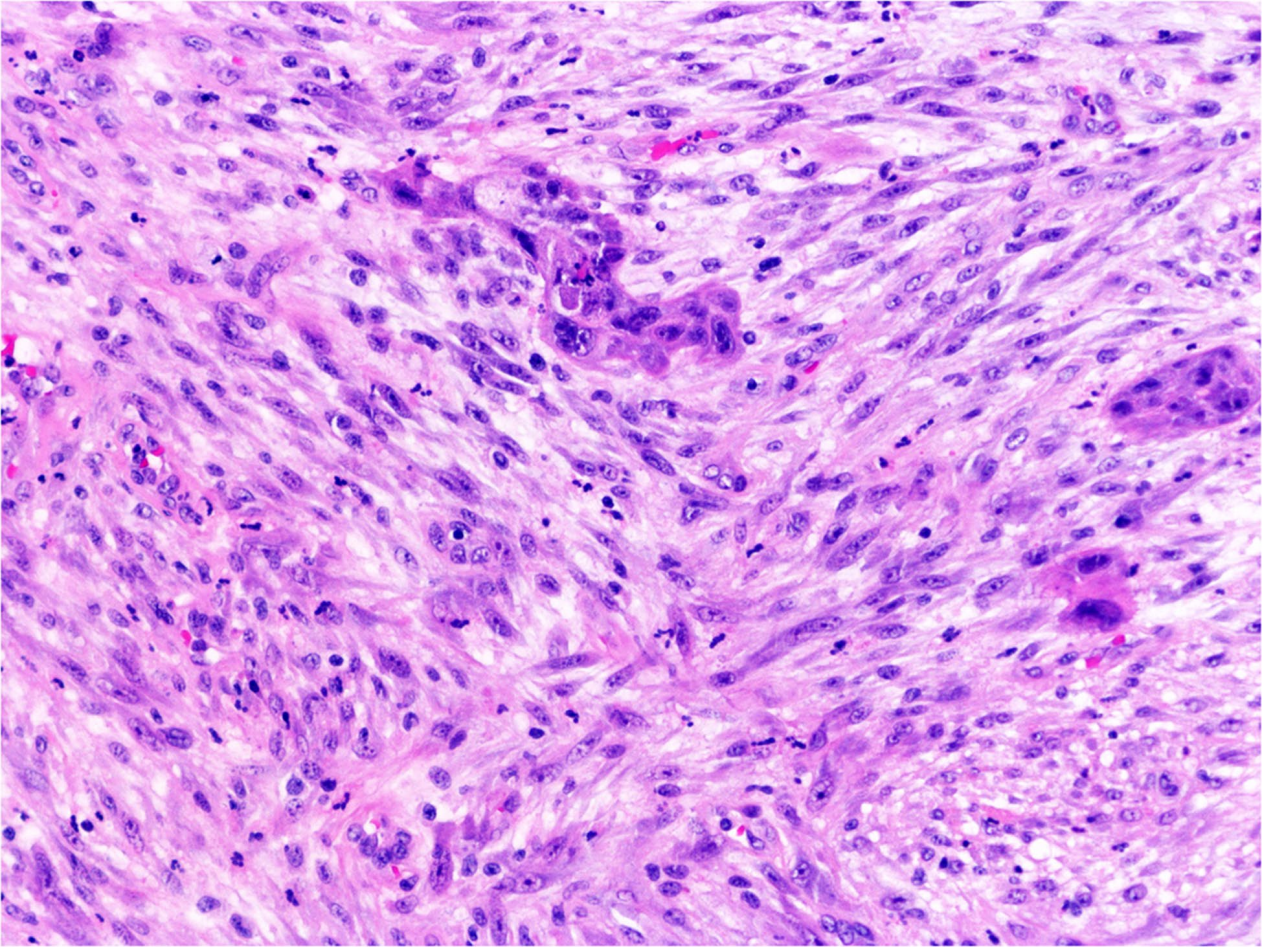
Spindle cell carcinoma: biphasic tumour, composed of islands of squamous cell carcinoma and malignant spindle cells

Macroscopically, SpCC can present either as an exophytic polypoid lesion or less frequently, as a flat, endophytic, or ulcero-infiltrative tumour [45, 46]. Microscopically, it can present as a biphasic tumour, with an invasive or in situ SCC component and a spindle cell component, in varying proportions (Fig. 4). SpCC can also consist only of spindle cells and may show heterologous differentiation, such as osseocartilagineous or rhabdomyoblastic differentiation [45, 47, 48]. Occasionally, it may be less cellular, with a prominent inflammatory infiltrate and reactive vessels with a granulation tissue-like appearance [49]. SpCC expresses cytokeratin, p63, p40, and CK5/6 in 48–83% of the cases, reaching a higher range with OSCAR immunohistochemistry. Spindle cells are always positive for vimentin and occasionally for other mesenchymal markers [42, 45, 50].

The diagnosis is straightforward when both spindle and SCC components are present. If only a spindle cell component is present, it must be differentiated from sarcomas and inflammatory myofibroblastic tumour [51], which are rare in the larynx. A malignant spindle cell tumour at this location is considered SpCC until proven otherwise. Granulation tissue–like SpCC must be distinguished from benign, reactive lesions which may be challenging due to the close clinical and histological overlap with several benign conditions. Franchi and Agaimy [49] described aberrant expression of p53 indicative of *TP53* mutations, consisting either of diffuse nuclear staining or absent (null) staining in SpCC cases with granulation tissue-like features, whereas all benign lesions showed weak to moderate nuclear p53 positivity.

Though originally suggested that SpCC is a more aggressive tumour than conventional SCC, follow-up studies of a large number of patients have challenged this common belief [52–55]. Gerry et al. [52] analysed 150 SpCCs of the larynx in comparison to 20,866 cases of SCC of the same locations, and Henock et al. [54] compared 155 SpCCs to 17 091 SCCs of the larynx. They found that the prognosis of SpCC of the larynx is comparable to or slightly better than conventional LSCC. Important prognostic factors seem to be previous radiation therapy, location, and macroscopic appearance. Patients with polypoid, exophytic SpCC with glottic origin, and no history of radiation exposure have a favourable prognosis, while those with flat, infiltrative lesions who had been treated with radiation prior to the development of SpCC have worse outcomes [46].

**Fig. 5:**
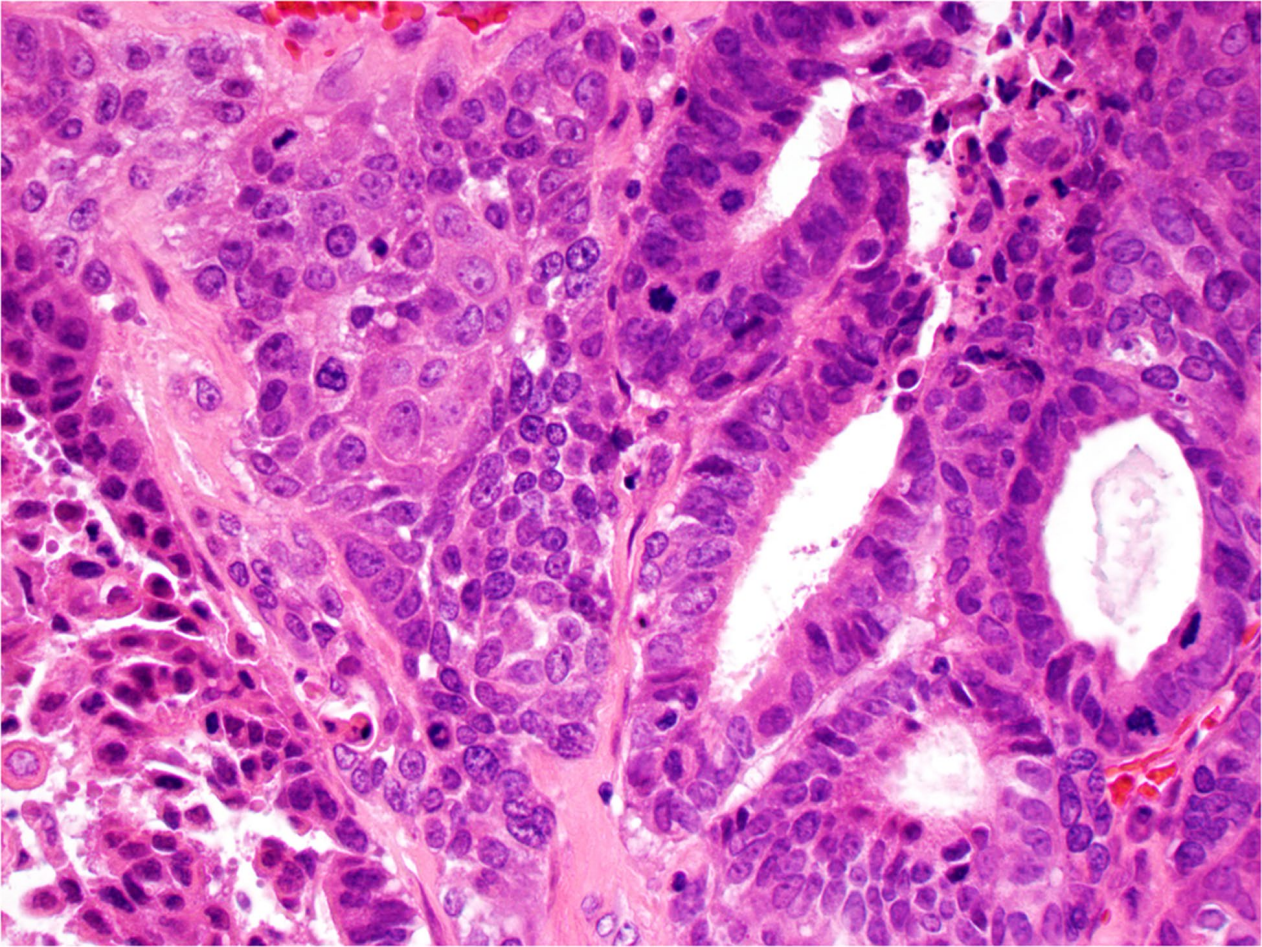
Adenosquamous carcinoma: biphasic tumour, composed of squamous cell carcinoma and adenocarcinoma in close proximity, but separate and distinct

*Adenosquamous carcinoma* (ASC) is a biphasic tumour that arises from the surface epithelium and shows squamous and glandular differentiation [56]. The SCC component is usually superficial, either as a carcinoma in situ or an invasive SCC, whereas the glandular component is usually located in a deeper part of the tumour, often exhibiting cribriform or tubular growth patterns (Fig. 5) [57]. The two components occur in close proximity and are generally distinct and separate or occasionally intermixed. Metastatic ASC may display one or both components. ASC is aetiologically related to smoking and alcohol consumption. Laryngeal ASC is not associated with HPV [58].

ASC must be distinguished from adenoid SCC and mucoepidermoid carcinoma, which have a better prognosis. The presence of mucin and true glandular spaces distinguishes ASC from adenoid SCS. Separate areas of squamous and glandular components and the presence of surface epithelial dysplasia or carcinoma in situ favour the diagnosis of ASC and not mucoepidermoid carcinoma. Molecular analysis might be helpful: *CRTC1/2::MAML2* gene fusions are characteristic of mucoepidermoid carcinoma. If present, the diagnosis of ASC can be excluded [59].

ASC has a worse prognosis than conventional SCC, usually presenting at an advanced stage, with frequent recurrences and dissemination despite surgical treatment with adjuvant chemo-radiotherapy. The median survival is < 3 years, and 5-year survival rates range between 30 and 50% [60, 61].

**Fig. 6:**
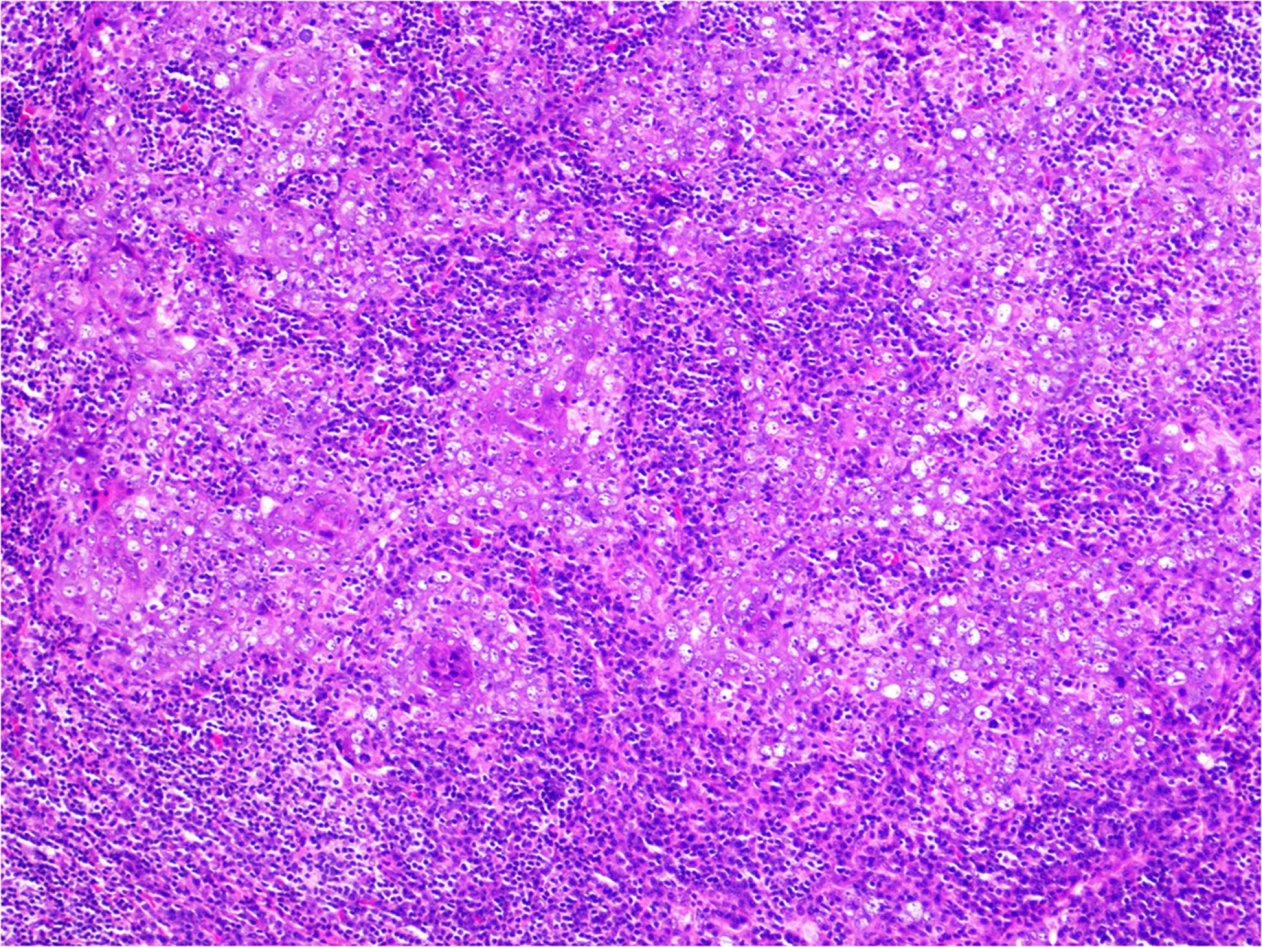
Lymphoepithelial carcinoma: poorly differentiated carcinoma accompanied by a prominent stromal lymphoplasmacytic infiltration

*Lymphoepithelial carcinoma* (LEC) is defined as a poorly differentiated SCC or undifferentiated carcinoma accompanied by a prominent stromal lymphoplasmacytic infiltration (Fig. 6). It is morphologically similar to nasopharyngeal carcinoma [62]. In contrast to nasopharyngeal SCC, laryngeal LEC is only rarely EBV-positive but may harbour high-risk HPV [63–65]. It expresses pan-cytokeratins and squamous markers (p40, p63, CK5/6). LEC must be distinguished from melanoma and lymphoma by proper immunohistochemistry, particularly when morphologic and immunohistochemical features of squamous differentiation are absent [62]. The prognosis is similar to conventional SCC, the 5-year disease-specific survival is approximately 65% [66, 67].

## NUT carcinoma

This is a rare, aggressive tumour characterised by cytogenetic aberrations involving the nuclear protein in testis gene *NUTM1* on chromosome 15q14. Though not considered a subtype of SCC by many authors, it often shows foci of squamous differentiation and is therefore an important differential diagnosis of SCC and poorly differentiated carcinoma [68]. The most common rearrangement in NUT carcinoma is a t(15;19) translocation resulting in a *BRD4::NUTM1* gene fusion*.* Several other *NUTM1* fusion partners have been identified, including *BRD3*, *NSD3*, *ZNF532*, and *ZNF592* [69, 70], and there is evidence suggesting that the fusion partner might be of clinical and prognostic relevance [71].

NUT carcinomas predominantly affect young adults but can occur at any age. They often arise from midline structures of the thorax and head and neck but can originate in almost any site. In the head and neck, NUT carcinoma accounts for 1% of all carcinomas and is usually located in the sinonasal tract, nasopharynx, or major salivary glands. It has also been described in the larynx [72–75]. NUT carcinoma is probably underdiagnosed, because its tumour phenotype overlaps with that of SCC or poorly differentiated carcinoma [71, 76].

**Fig. 7:**
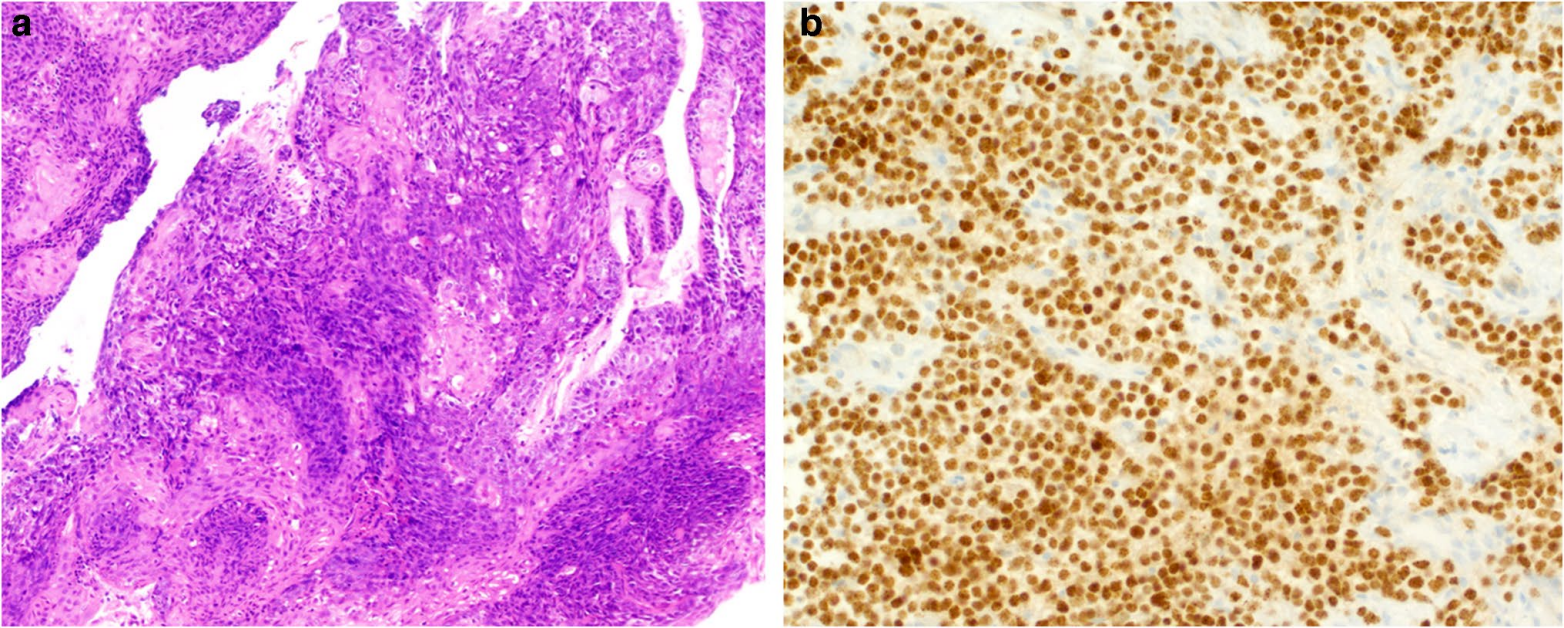
NUT carcinoma: poorly differentiated carcinoma with focal squamous differentiation (**a**) and diffuse nuclear staining for NUTM1 protein by immunohistochemistry (**b**)

Microscopically, it is a poorly differentiated carcinoma, consisting of a relatively uniform population of small to intermediate-sized undifferentiated cells. In one-third of the cases, there are abrupt foci of keratinisation or squamous differentiation (Fig. 7a). High mitotic activity, numerous apoptotic bodies, and areas of necrosis are typically present. However, these morphologic features are not specific to NUT carcinoma [68, 70, 72, 77].

The differential diagnoses include conventional SCC, and if no squamous differentiation is present, also neuroendocrine carcinoma, small round-cell sarcoma (e.g. Ewing sarcoma, CIC-rearranged sarcoma), haematolymphoid neoplasm (e.g. lymphoma, myeloid sarcoma), melanoma, and germ cell tumours [68].

The diagnosis is based on the identification of *NUTM1* gene rearrangements by FISH, RT-PCR, or NGS methods, but importantly, diffuse (> 50%) nuclear staining for the NUTM1 protein (Fig. 7b), usually with a characteristic speckled appearance, is considered sensitive and specific enough to support the diagnosis [78]. NUT carcinoma also shows positive staining for cytokeratins, p63, and CD34 (in approximately half of the cases) and is negative for S100, HMB45, desmin, myoglobin, smooth muscle actin, muscle actin, chromogranin, and synaptophysin [68]. However, p63/p40 negative cases occur and might be missed if not thought of.

NUT carcinoma is an aggressive tumour, with a median survival of only 6–9 months. Approximately 70–80% of diagnosed patients die within 2 years. Patients with non-*BRD4*::*NUTM1* fusions (*BRD3*::*NUTM1* or *NSD3*::*NUTM1*) have significantly better survival (median overall survival time, 36.5 months) than those with the classical *BRD4*::*NUTM1* fusion (median overall survival time, 10 months) [79].

There is currently no consensus on standard therapy for NUT carcinoma. The extent of surgical resection and initial radiotherapy has been reported as independent predictors of survival [80–82]. Long-term survival has been reported in a few patients receiving an aggressive combined approach with radiotherapy and surgery [12, 83, 84].

## HPV-associated squamous cell carcinoma of the larynx

The role of HPV in SCC largely depends on the anatomic site. In contrast to the oropharynx, HPV-associated SCC is less common in the larynx. A wide range of HPV infection rates has been reported in the larynx, ranging from 1.6 to 83% [85]. The infection rates depend on the methodology used for HPV detection and vary among geographical regions, being high in China and low in Europe and North America [85]. Approximately 20% of LSCCs are associated with HPV [8]. The majority of positive LSCCs are associated with high-risk HPV (HPV 16/18) and rarely with low-risk HPV (HPV 6/11) [83, 86]. Interestingly, a rising incidence or proportion of HPV-associated SCC in all subsites of the head and neck has been reported [87].

Various methods for HPV detection are nowadays available. mRNA in situ hybridization (ISH) is considered the gold standard for the detection of HPV-associated carcinoma. p16^INK4a^ immunohistochemistry, with a nuclear and cytoplasmic 70% cut-off positivity has been widely used as a surrogate marker for HPV-associated carcinoma of the oropharynx [88, 89]. However, p16 immunohistochemistry as a surrogate marker for HPV-associated SCC may be less reliable in the larynx and hypopharynx than in the oropharynx [90, 91]. A higher proportion of LSCC overexpresses p16 but is HPV-independent compared with other head and neck sites [90, 92, 93]. In a study by Mena et al. [90], the percentage of concordance between positive p16 immunohistochemistry (cut-off ≥ 70%) and E6*I mRNA among HPV-DNA-positive oropharyngeal SCC was 82.1% but only 56.9% in LSCC. The possible prognostic significance of p16 positivity in HPV-independent LSCC remains to be determined [91, 94].

The clinical implication of HPV in LSCC is not clear. Early studies suggested that HPV does not affect the prognosis at this site [95–98]. However, recent studies have provided conflicting results suggesting that HPV-associated LSCC may have a better prognosis [94, 99–105]. Meta-analyses by Shi et al. [103] and Sahovaler et al. [94] including 18 and 24 primary investigations, respectively, showed an improved survival for HPV-associated LSCC. HPV-associated SCC of the head and neck, when grouped across five non-oropharyngeal subsites had a better 5-year overall survival than their HPV-independent counterparts (62.9% vs 54.7% [87]. A more significant improvement in survival was found for HPV-associated SCC of the hypopharynx than larynx [87].

Microscopically, HPV-associated LSCC exhibits equivalent features as its prototypic non-keratinising HPV-associated oropharyngeal counterpart [101]. Occasionally, it presents as papillary SCC [39, 40, 106] or lymphoepithelial carcinoma [65]. Recently, a novel morphologic subtype of HPV-associated SCC of the larynx and hypopharynx was described. It is characterised by warty morphology with exophytic growth, prominent surface keratosis and parakeratosis, koilocytosis, and nuclear pleomorphism [101]. Similar morphologic features have been described in HPV-associated anogenital SCC. It tends to present at an early stage, with a trend towards favourable outcomes [101].

Interestingly, precancerous lesions have been found in the larynx in association with high-risk HPV infection. They are characterised by the proliferation of atypical basaloid cells with surface parakeratosis, along with p16 immunoreactivity and the presence of transcriptionally active HPV, which can be best highlighted by E6/E7 RNA in situ hybridization [108, 109].

Though the body of evidence is still limited, more frequent testing for HPV in LSCC may be justified and could provide new information on whether HPV status should be incorporated in the prognostication of patients with LSCC. HPV testing should particularly be performed in LSCC cases with basaloid, papillary, lymphoepithelial, or warty morphology [101, 107].

## Histopathology assessment of squamous cell carcinoma of the larynx

The majority of LSCCs are moderately differentiated keratinising tumours, and the diagnosis is usually straightforward. Poorly differentiated tumours may require ancillary studies for better further subclassification (NUT carcinoma, basaloid SCC, spindle cell SCC) and to be distinguished from neuroendocrine neoplasms, melanoma, lymphomas, and sarcomas (rhabdomyosarcoma, leiomyosarcoma, malignant peripheral nerve sheath tumour).

The microscopic assessment of SCC has not significantly changed, as it is still graded according to the modified Broder’s criteria into well, moderately, and poorly differentiated SCC, with a limited prognostic significance. There are many pathologic features which have been traditionally associated with poor prognosis and must be included in the routine pathology report: SCC type (conventional vs SCC subtypes), site affected, size, focality, tumour grade, the presence of lymphovascular and perineural invasion, extent of invasion, margin status, nodal status, and extranodal extension in lymph node metastases [110, 111].

Importantly, pTNM assessment for LSCC is demanding as it is based on a combination of clinical and morphological criteria. Clinical information is needed, i.e. the mobility of the vocal cord, to correctly assess the pT status [112, 113]. Moreover, pT correlates poorly with prognosis, particularly in patients with advanced cancer: patients with pT4aN0 have been shown to have better survival than patients with pT3N + [114, 115] suggesting an independent effect of T and N stages on survival in LSCC [116]. The assessment of the primary tumour and the criteria for pT in LSCC as they are defined in the current staging system may have a poor prognostic value. Future studies will show whether there are other biomarkers to be added to the pathology report that may help to better predict outcomes.

## Depth of invasion: a new parameter for patients’ risk stratification?

Tumour size, including the largest tumour dimension and thickness, is generally regarded as important in cancer. Specifically, depth of invasion (DOI) has been shown to be closely associated with patients’ survival in many cancer types. To measure DOI, the basement membrane adjacent to the carcinoma is identified, and an imaginary line is drawn following the contour of the “normal” anatomy. A vertical or “plumb line” from that line that extends to the deepest part of the tumour represents the DOI. DOI stratifies tumours better than tumour thickness. It enables us to distinguish exophytic tumours which may be thicker than ulcerative tumours but have a smaller DOI and a better prognosis than ulcerative tumours which may be thinner but more deeply infiltrative. In the head and neck, DOI has been widely studied as an indicator of poor prognosis, especially in oral SCC, where it is now included as a criterion for pT assessment in UICC/AJCC staging classifications [112, 113].

The tumour dimension is currently not part of the pathologic staging of LSCC [11, 12], whereas clinical evidence of cord fixation (paralysis) is incorporated instead. However, there is emerging evidence suggesting that DOI is also an important prognostic factor in LSCC. Studies of total or partial laryngectomy specimens have suggested that DOI correlates with the presence of perineural invasion, lymphovascular invasion, and nodal metastasis [117–120] and that it can be reliably assessed preoperatively using CT imaging [121]. Wang et al. [120] reported DOI as an independent factor influencing overall survival and relapse-free survival in patients with LSCC. Furthermore, the anatomical location of LSCC is widely accepted as an important parameter, with differences in the biological characteristics of invasion and metastasis of supraglottic, glottic, and subglottic SCC. Importantly, Wang et al. [120] found that patients with DOI less than 3.5 mm showed a good prognosis in both supraglottic and glottic SCC. With an increase in DOI, glottic SCC may spread from the parallel space to the supraglottic space. Supraglottic SCC will also invade the paraglottic space or even subglottic space with an increase in DOI. Therefore, different subtypes of LSCC may confound the anatomical classification after reaching a certain DOI, resulting in a homogenous biological outcome. The traditional staging method may overlook this point. These findings suggest DOI to be considered part of future pathological UICC/AJCC staging for LSCC, enabling better risk stratification and improved treatment decisions for LSCC [120].

## Lymphovascular invasion: should it be stratified as lymphatic and vascular, and extratumoural and intratumoural?

Reports on the prognostic value of lymphovascular invasion (LVI) in LSCC are variable, but some studies of total or partial laryngectomy specimens suggest that it is an independent indicator of poor outcome [122–125]. LVI includes invasion of lymphatic and blood vessels of various sizes. With small calibre vessels, it is often difficult to distinguish among the vessel types, and their invasion by tumour cells is usually reported as LVI.

With invasion of medium- to large-sized vessels, vascular and lymphatic invasion can be distinguished and reported as venous invasion which is recognised by the presence of tumour cells within an endothelium-lined space surrounded by a rim of smooth muscle and containing red blood cells, or lymphatic invasion recognised by the presence of tumour cells in thin-walled structures lined by endothelium, without an identifiable smooth muscle layer or elastic lamina.

Recognition of LVI may be difficult and subjective and can be improved by using immunohistochemistry highlighting endothelial cells with CD31, CD34, ERG, and FLI-1 or specifically highlighting lymphatic endothelium with D2-40 [126] and histochemical stains (e.g. elastic staining to identify venous elastic lamina). However, these methods are not widely used in routine work. In thyroid neoplasms, CD61 has been shown as a promising novel marker as it is a marker of activation of the fibrinogen cascade, and its presence in a linear fashion on platelets in association with tumour cells in a vessel space confirms that fibrin is present, and thus, genuine endothelial destruction is present [126].

It is currently not clear whether subtyping LVI according to vessel type and size might be prognostically significant. Similarly, the significance of extratumoural vs intratumoural LVI remains to be determined in LSCC. However, it has been shown to have important prognostic value in some cancers, for example in colorectal carcinoma [127]. Therefore, further evaluation of these parameters is warranted.

## Perineural invasion: do nerve size and location matter?

Perineural invasion (PNI) is generally regarded as a poor prognostic feature in head and neck cancer including LSCC. It has been associated with high recurrence rates and poor survival [122, 128–139]. Brandwein-Gensler et al. [132, 139] considered PNI to be an important component of the risk assessment model to predict local recurrence and overall survival in head and neck SCC. Involvement of the major nerves (> 1 mm) was significantly associated with poorer outcomes as compared with PNI of small nerves or tumours with no PNI and has been given a score of three, directly placing these patients in the high-risk category for loco-regional recurrence.

There are several characteristics which might further categorise the impact of PNI on prognosis: extratumoural vs intratumoural PNI, the number of PNI foci, the calibre of the largest involved nerve, involvement of a large-calibre or a “named” nerve, and intraneural invasion [128, 131, 132, 140–144].

Previous studies have demonstrated that PNI in LSCC affects minor nerves whereas superior and recurrent laryngeal nerves are not involved [137, 145, 146]. These findings suggest that perineural tumour spread through superior and recurrent laryngeal nerves does not constitute a pathway of spread in LSCC, even in locally advanced cancer. Therefore, there is no need to extend the surgical resection boundaries in total laryngectomy to include the bilateral superior and recurrent laryngeal nerves [137].

**Fig. 8:**
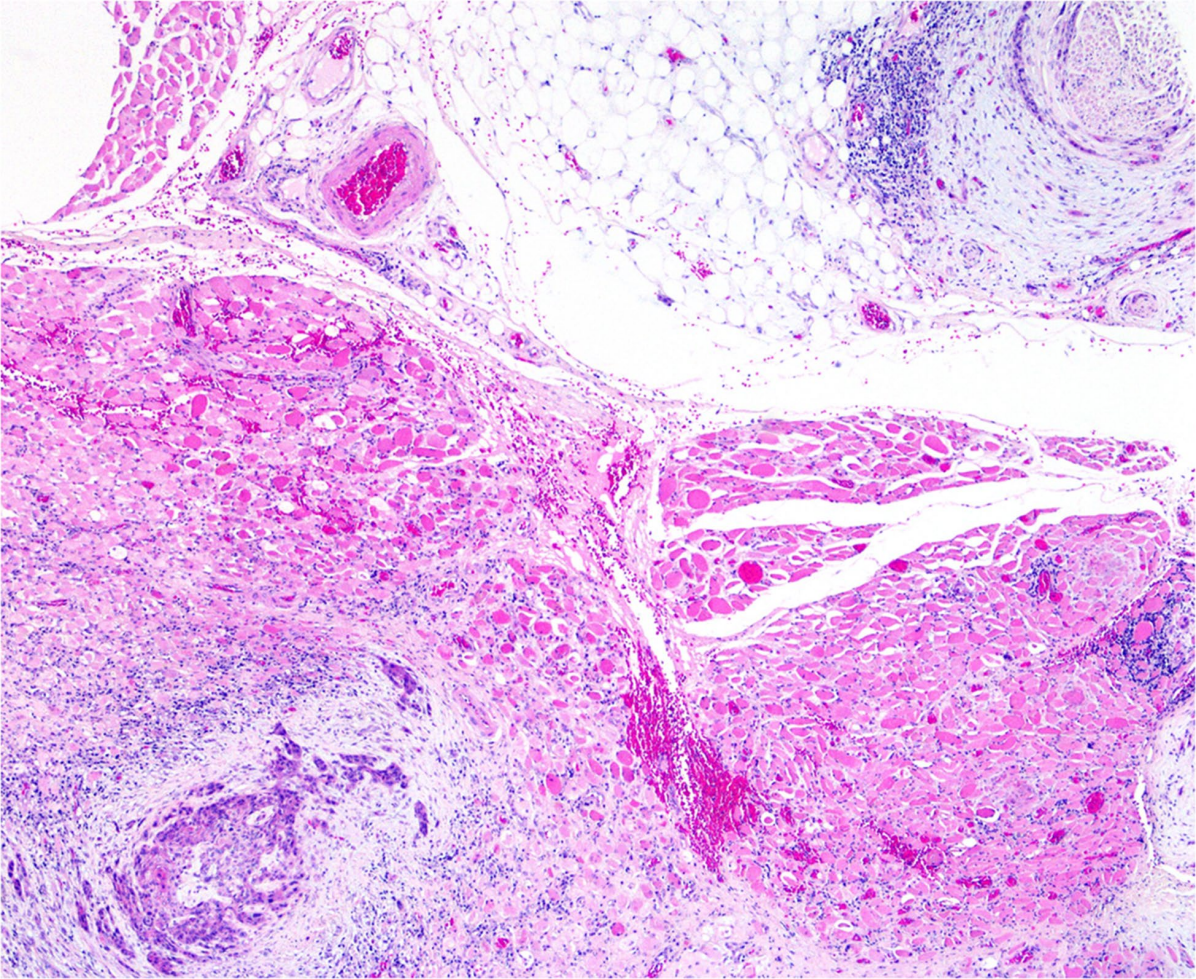
Extratumoural perineural invasion (upper right corner), 2 mm away from the deepest part of the tumour (lower left corner)

Extratumoural PNI (Fig. 8) might be more important than intratumoural PNI [131], but there is currently insufficient evidence to separate PNI into extratumoural and intratumoural invasion. Additional studies evaluating these parameters may further shed prognostic significance on the patient outcomes.

## Tumour budding: time to include it in the report, but how to assess it?

**Fig. 9:**
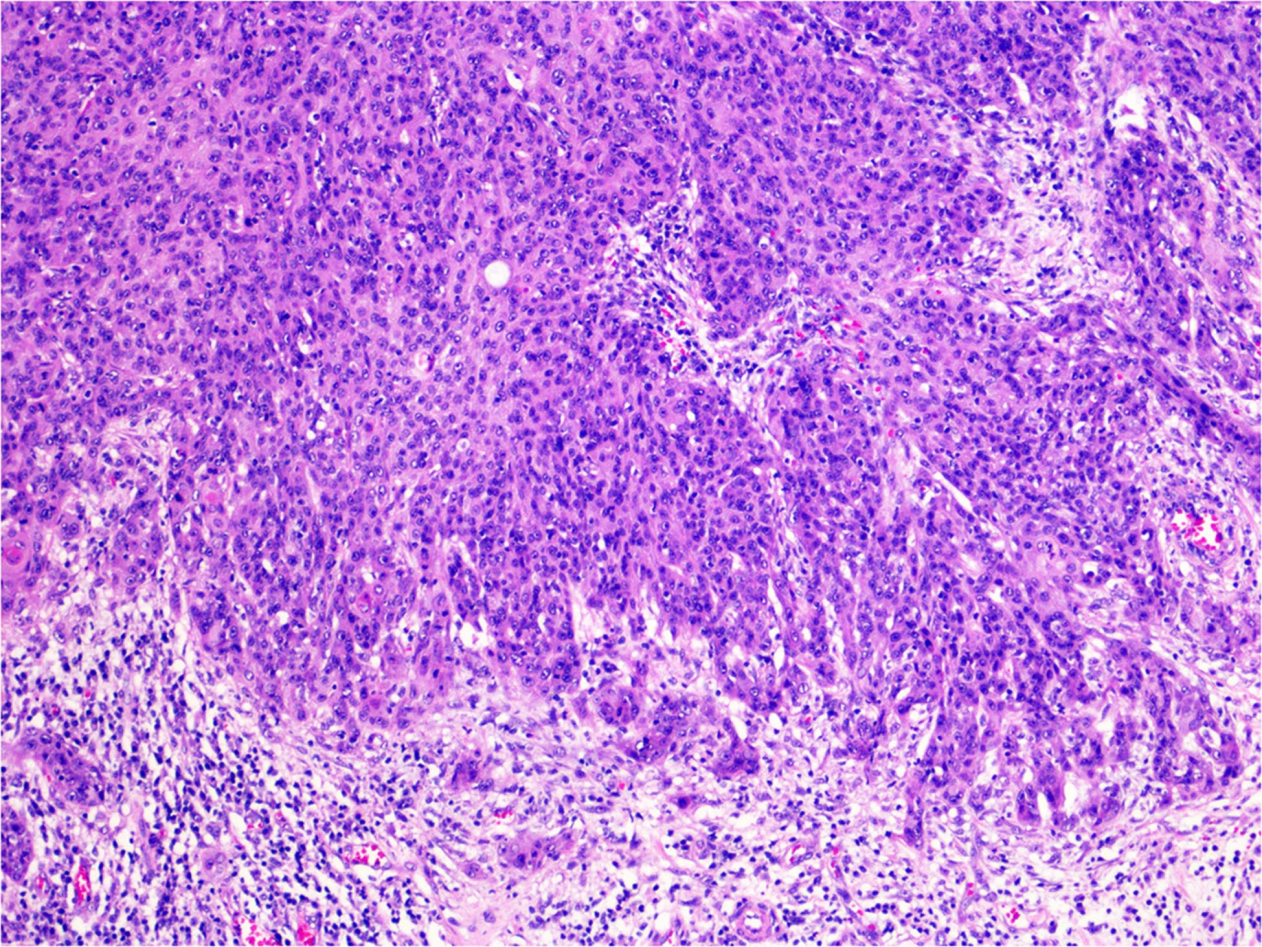
Tumour budding: single tumour cells or clusters of up to four tumour cells at the invasive tumour front

The pattern of invasion at the invasive tumour front is an important prognostic parameter in many cancer types including head and neck SCC [147–149]. In particular, the presence of tumour budding at the invasive tumour front has emerged as a promising biomarker. Tumour budding is usually defined as single tumour cells or clusters of up to four tumour cells at the invasive tumour front (Fig. 9) [150]. There is evidence suggesting that it is an independent adverse prognostic factor in head and neck SCC including LSCC, associated with an increased risk of lymph node metastasis and worse overall survival [151–161].

Even though budding is a promising biomarker in head and neck SCC, there is no consensus yet on how it should be assessed and graded [161, 162]. It has been recommended to count the number of buds in high power fields in areas showing maximal budding [152]. Depending on the eyepiece field diameter of the microscope, the number of buds may need to be normalised to represent the number for a field of 0.785 mm^2^ (20 × objective with an eyepiece diameter of 20 mm). It remains to be defined whether budding assessment should be performed on HE slides or using immunohistochemistry for cytokeratins, and what the cut-off values for grading budding activity are. A three-tier scoring system (low risk ≤ 4 buds, intermediate risk 5–9 buds, high risk ≥ 10 buds) can be used, but most of the studies recommend adopting a two-tier scoring system with a cut-off point of five buds (low risk < 5 buds vs. high risk ≥ 5 buds). Generally, the ≥ 5 buds cut-off seems to be an appropriate discriminator to stratify patients [163] without ancillary testing employed.

## Tumour‐infiltrating lymphocytes

Tumour-infiltrating lymphocytes (TILs) in the tumour microenvironment have been recognised as an important biomarker in a variety of malignancies including head and neck SCC [164, 165]. Rodrigo et al. [166] performed a meta-analysis of publications investigating TILs in LSCC and confirmed its prognostic significance. High infiltration of TILs was reported to be associated with a more favourable clinical course and to correlate with PD-L1 expression [149, 167–173]. The combination of high CD8^+^ TIL infiltration and positive PD-L1 expression, also referred to as type I tumour microenvironment or adaptive immune resistance [174], confers a favourable prognosis in patients with head and neck SCC, indicating that this is the most favourable immune microenvironment to mediate effective host immune responses that can restrain tumour growth. Moreover, patients with type I tumour immune microenvironment are likely to benefit from therapy with immune checkpoint inhibitors [170, 171]. Available data thus indicate that TILs should be considered a standardised and validated biomarker in the routine pathology report of LSCC patients.

## Open approach surgery vs endoscopic surgery, early vs advanced carcinoma

Surgery remains the mainstay of treatment for LSCC. In addition to total laryngectomy, organ-preserving surgical techniques including transoral laser microsurgery have been successfully introduced. They are associated with excellent outcomes, preservation of laryngeal function and avoidance of radiotherapy complications if patients are selected correctly [175–178].

The specimens obtained by endoscopic surgery differ significantly from those obtained by open-approach surgery: they are small, with crush and thermic artefacts and a limited amount of surrounding tissue, often with inflammation and scarring due to previous biopsies. Despite the fact that interpretation of this type of biopsy specimens is challenging, reliable prognostic and predictive factors would be most helpful to guide the need for additional treatment. Moreover, it is often difficult to prove invasive SCC, so additional markers of invasion vs dysplasia and in situ carcinoma are needed. In the case of invasive SCC, DOI and tumour budding seem promising as potential predictive factors. On the other hand, some predictive markers can only be assessed properly and might prove useful in total or partial laryngectomy specimens, e.g. extratumoural PNI and LVI.

## Conclusion

Despite significant progress in the last decades, LSCC is still associated with significant morbidity and mortality worldwide. New biomarkers are therefore needed to enable a more personalised treatment approach and to predict unfavourable clinical course. We summarised histopathologic features that are already assessed routinely (e.g. subtypes of LSCC) as well as those that are still of controversial prognostic significance (e.g. HPV-association, extratumoural vs intratumoural LVI and PNI, tumour budding, DOI, TILs).

Further studies are warranted to see which of the described features may help to better stratify patients with LSCC. Their introduction to routine work would be demanding as LSCC is characterised by complex anatomy and various surgical approaches. Separate guidelines will be required for the histopathologic examination of endoscopic surgery vs open-approach surgery specimens, describing the list of diagnostic, prognostic and predictive factors to be reported.

## Data Availability

Data supporting the findings of this study are available within the article. The complete datasets generated duringand/or analyzed during the current study are available from the corresponding author upon reasonable request.
