# Correction to: The impact of histopathology on prognosis of squamous cell carcinoma of the larynx: can we do better?

**DOI:** 10.1007/s00428-025-04186-3

**Published:** 2025-07-28

**Authors:** Nina Zidar, Lester D. R. Thompson, Abbas Agaimy, Göran Stenman, Henrik Hellquist, Alfons Nadal, Antti A. Mäkitie, Fernando López, Primož Strojan, Alfo Ferlito

**Affiliations:** 1 Faculty of Medicine, Institute of Pathology, University of Ljubljana, Korytkova 2, 1000 Ljubljana, Slovenia; 2 Head and Neck Pathology Consultations, Woodlands Hills, CA USA; 3 Comprehensive Cancer Center (CCC) Erlangen-EMN, Institute of Pathology, University Hospital, Friedrich-Alexander University Erlangen-Nürnberg (FAU), Erlangen, Germany; 4 Department of Pathology, Sahlgrenska Center for Cancer Research, Sahlgrenska University Hospital, University of Gothenburg, Gothenburg, Sweden; 5 Department of Biomedical Sciences and Medicine, ABC-RI, University of Algarve, Faro, Portugal; 6 Department of Cellular Pathology, Northern Lincolnshire and Goole NHS Foundation Trust, Lincoln, UK; 7 Department of Pathology, Hospital Clinic, Barcelona, Spain; 8 Department of Basic Clinical Practice, School of Medicine, Universitat de Barcelona, Barcelona, Spain; 9 Department of Otorhinolaryngology-Head and Neck Surgery, Research Program in Systems Oncology, Helsinki University Hospital, University of Helsinki, Helsinki, Finland; 10 Department of Otolaryngology, ISPA, IUOPA, CIBERONC, Hospital Universitario Central de Asturias, University of Oviedo, Oviedo, Spain; 11 Department of Radiation Oncology, Institute of Oncology, Ljubljana, Slovenia; 12 University of Udine School of Medicine, Udine, Italy

**Correction to** **:** **Virchows Archiv**

10.1007/s00428-025-04082-w

In the published paper, Fernando López's given name and family name were swapped.

The original article has been corrected.

